# Energy metabolism in cardiovascular diseases: unlocking the hidden powerhouse of cardiac pathophysiology

**DOI:** 10.3389/fendo.2025.1617305

**Published:** 2025-06-05

**Authors:** Li Chen, Mingtai Chen, Xinrui Yang, Yuanli Hu, Caiwei Qiu, Youyou Fu, Xiaoyu Lan, Gang Luo, Qiuyu Liu, Mengnan Liu

**Affiliations:** ^1^ The Affiliated Traditional Chinese Medicine Hospital, Southwest Medical University, Luzhou, Sichuan, China; ^2^ Department of Cardiovascular Disease, Shenzhen Traditional Chinese Medicine Hospital, Shenzhen, Guangdong, China; ^3^ School of Pharmacy, Southwest Medical University, Luzhou, China

**Keywords:** cardiovascular diseases (CVDs), energy metabolism, metabolic reprogramming, mitochondrial dysfunction, precision therapeutic targets

## Abstract

Cardiovascular diseases (CVDs) remain the leading cause of global mortality, yet their pathogenesis has not been fully elucidated, particularly regarding the role of abnormal energy metabolism. Major outstanding questions pertain to the dynamic regulation of metabolic reprogramming and its complex interplay with mitochondrial dysfunction. Previous studies have demonstrated that the heart, as a high-energy-demand organ, relies on the dynamic equilibrium of substrates such as fatty acid (FA) and glucose to sustain adenosine triphosphate (ATP) production. Metabolic disturbances—characterized by suppressed FA oxidation and aberrant activation of glycolysis—directly contribute to the pathological progression of various CVDs, including heart failure (HF), atherosclerosis, and myocardial infarction(MI), through mechanisms involving oxidative stress, inflammatory responses, and an energy crisis. This review systematically examines the core pathways of cardiac energy metabolism (e.g., mitochondrial oxidative phosphorylation (OXPHOS), regulation of glucose and lipid metabolism) and their dysregulation in disease states, while evaluating intervention strategies targeting metabolic pathways, such as mitochondrial function enhancement and substrate utilization modulation. Future research directions emphasize the integration of metabolomics with clinical translational studies to comprehensively decipher the multidimensional regulation of metabolic networks, thereby facilitating the development of novel precision therapeutic targets.

## 1 Introduction

Cardiovascular diseases (CVDs), encompassing ischemic heart disease, atherosclerosis, heart failure (HF), and coronary artery disease, constitute the predominant cause of global mortality and disability (1, 2). A hallmark of cardiac energy metabolism lies in its dynamic substrate utilization capacity (termed metabolic reprogramming), which manifests as adaptive adjustments of energy metabolic pathways under distinct pathological conditions—such as the shift from FA oxidation to glucose oxidation in HF and glycolytic activation in atherosclerosis—thereby serving as a critical intervention target for CVD prevention and treatment. Notably, metabolic dysregulation represents an early pathological feature of multiple CVDs, yet its precise molecular mechanisms remain incompletely defined (3, 4). Thus, elucidating the contributions of energy metabolism to CVD pathogenesis—especially myocardial mitochondrial dysfunction and the spatiotemporal control of metabolic pathways—remains imperative. Interventions targeting metabolic reprogramming have emerged as a pivotal research direction to overcome current therapeutic limitations. Current clinical management strategies primarily include surgical interventions, pharmacological therapies, and non-pharmacological approaches such as lifestyle modifications (5, 6). While CVD risk factors are categorized into non-modifiable elements (e.g., age, sex, genetic background) and modifiable components (including smoking, dietary imbalances, hypertension, type 2 diabetes, and dyslipidemia), incomplete elucidation of CVD pathogenesis continues to impede the development of novel therapies.

Substantial knowledge gaps persist in understanding the molecular mechanisms underlying cardiomyocyte mitochondrial dysfunction and the spatiotemporally regulated metabolic networks. These limitations contribute to the suboptimal specificity and uncertain long-term efficacy of current metabolic modulators, such as AMP-activated protein kinase(AMPK) activators and mitochondria-targeted antioxidants. Although suppressing FA oxidation (FAO) or enhancing glucose metabolism may transiently improve cardiac function, the signaling mechanisms of metabolic intermediates and the causal relationship between aberrant mitochondrial dynamics and disease phenotypes remain to be systematically investigated. To address these challenges, integrating cutting-edge technologies like metabolomics and single-cell sequencing to precisely delineate the dynamic regulatory networks of cardiac metabolic reprogramming, coupled with developing therapies targeting critical metabolic nodes, represents a core challenge in advancing precision medicine for CVDs. This review focuses on elucidating the molecular interplay between energy metabolism dysregulation and CVD progression, while systematically exploring novel therapeutic strategies targeting metabolic pathways, with the ultimate goal of providing theoretical foundations and innovative directions to transcend the limitations of conventional treatments.

## 2 Cardiac energy metabolism

### 2.1 Energy sources and metabolic substrates in the heart

**Figure 1:**
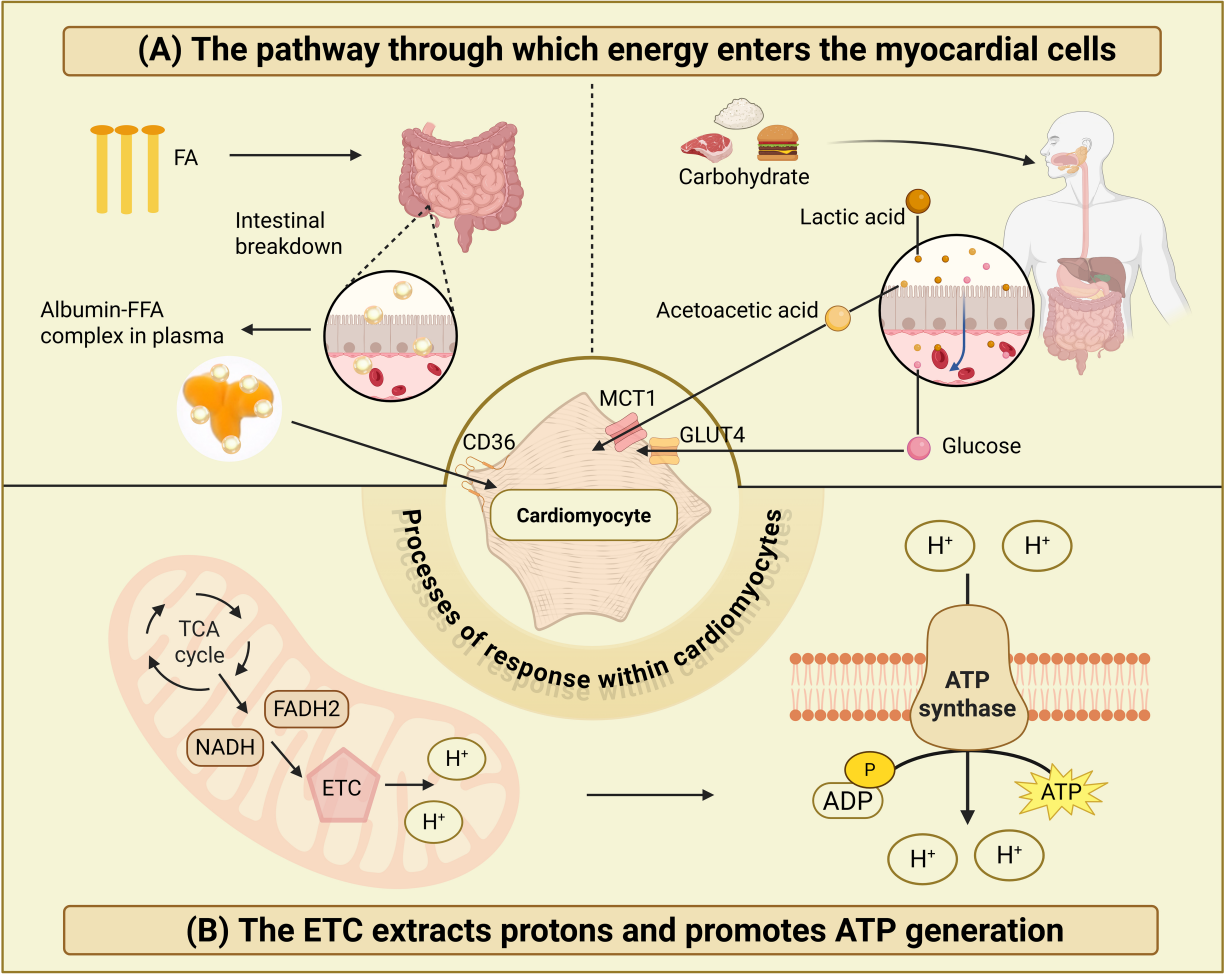
Cardiac substrate utilization and ATP production pathways. **(A)** The pathway through which energy enters the myocardial cells. **(B)** The ETC extracts protons and promotes ATP generation. **Figure 1** illustrates cardiac substrate utilization and ATP production pathways. Panel **(A)** depicts pathways through which energy substrates enter cardiomyocytes via specific membrane transporters. Panel **(B)** demonstrates how mitochondrial ETC utilizes NADH and FADH_2_ derived from TCA cycle oxidation to establish a proton gradient that drives ATP synthase-mediated ATP production. Both panels strictly correspond to the described biochemical processes without extraneous additions.

In the adult heart, FA serve as the primary energy source, with additional contributions from glucose, ketone bodies, and amino acids. FAs are predominantly derived from the digestion and absorption of dietary lipids. Following intestinal processing, free FAs bind to plasma albumin and are transported into cardiomyocytes via membrane-associated transporters such as CD36 (7, 8). Glucose, originating from dietary carbohydrates, enters the bloodstream post-absorption and is internalized into myocardial cells through glucose transporters type 1 and type 4 (GLUT1/GLUT4) (9). Other substrates, including amino acids, lactate, and ketone bodies, are imported via specific membrane transporters such as monocarboxylate transporter 1 (MCT1) (10). These substrates are metabolized through the tricarboxylic acid (TCA) cycle, undergoing complete oxidation to generate reduced nicotinamide adenine dinucleotide (NADH) and flavin adenine dinucleotide (FADH2). These coenzymes drive the mitochondrial electron transport chain (ETC) to establish a proton gradient, which ultimately powers adenosine triphosphate (ATP) synthase to produce ATP, as illustrated in **Figure 1**.

Under normoxic conditions, oxidative phosphorylation (OXPHOS) accounts for approximately 95% of ATP synthesis, with FAs (40–60%) and glucose (20–40%) serving as the predominant substrates. In contrast, hypoxia shifts energy production toward glycolysis and lactate metabolism (10, 11). During intrauterine development, the fetus exists in a relatively hypoxic environment, predominantly relying on glycolysis and lactate metabolism for energy production. Postnatal adaptation in neonates is marked by a gradual transition toward glucose oxidation as the primary cardiac energy supply mode, owing to increased oxygen availability (12, 13). In contrast, the aging heart exhibits diminished metabolic flexibility: mitochondrial functional decline leads to reduced FAO efficiency, attenuated AMPK/SIRT1 signaling exacerbates oxidative stress and lipid accumulation, and impaired autophagy—particularly mitophagy—further aggravates lipotoxicity and energy crisis (14). These age-dependent metabolic shifts underscore the dynamic nature of cardiac energy adaptation across the lifespan, highlighting critical molecular nodes for therapeutic intervention in age-related cardiovascular pathologies. Cardiac energy metabolism involves intricate competition and regulatory interplay among substrates. For instance, enhanced FA oxidation suppresses glucose oxidation, mediated by inhibitory effects of FA-derived intermediates or metabolic regulatory mechanisms on enzymes or pathways critical to glucose utilization (11). This dynamic substrate utilization capability enables the heart to precisely adapt energy substrate preferences to meet physiological and pathological demands, ensuring optimal ATP supply.

### 2.2 Mitochondrial oxidative phosphorylation

**Figure 2:**
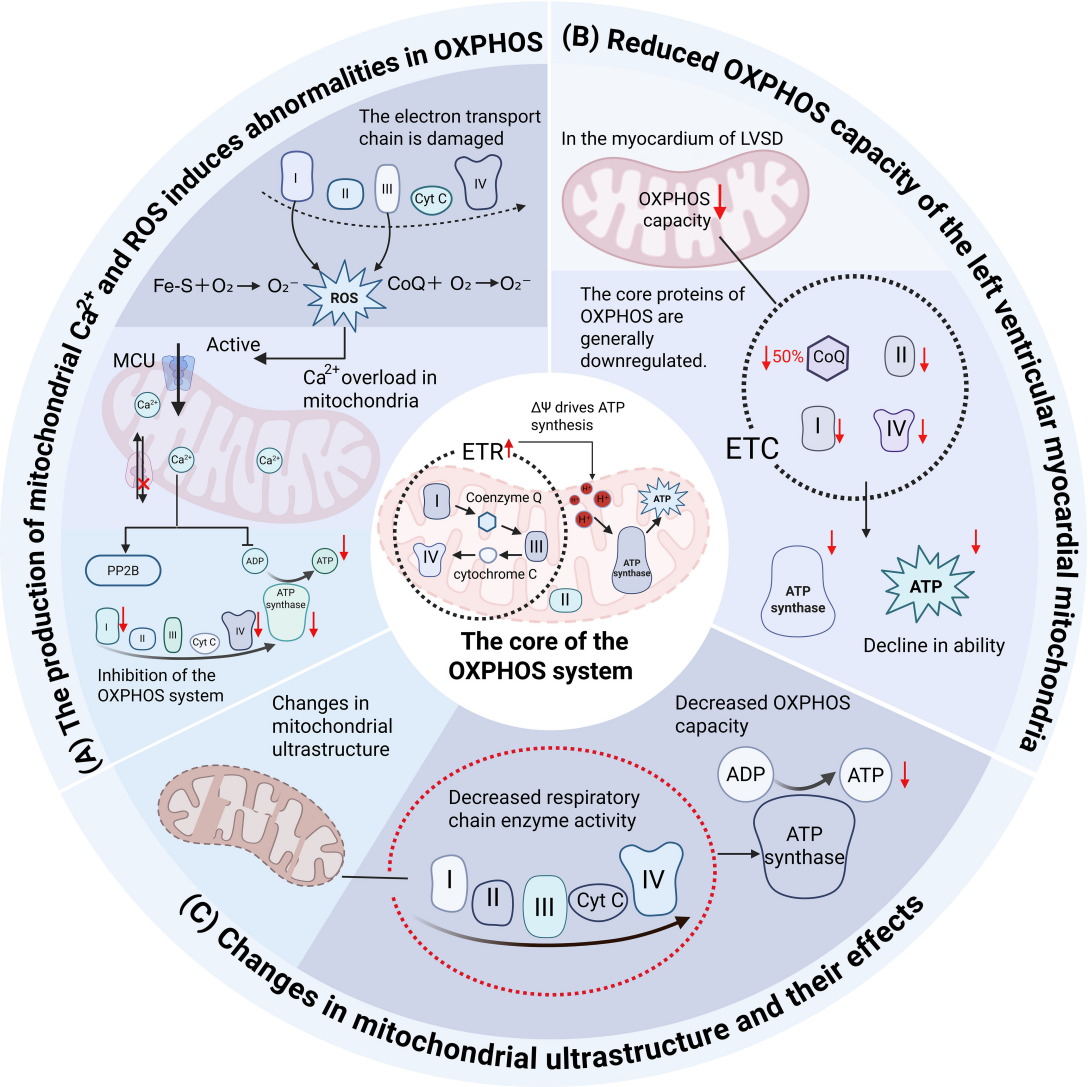
Mitochondrial dysfunction-induced oxidative stress and metabolic dysregulation. **(A)** The production of mitochondrial Ca^2+^ and ROS induces abnormalities in OXPHOS. **(B)** Reduced OXPHOS capacity of the left ventricular myocardial mitochondria. **(C)** Changes in mitochondrial ultrastructure and their effects. **Figure 2** delineates mitochondrial dysfunction-induced oxidative stress and metabolic dysregulation in cardiovascular diseases. Panel **(A)** illustrates how mitochondrial Ca²^+^ overload and reactive oxygen species (ROS) production impair oxidative phosphorylation (OXPHOS) by disrupting electron transport chain (ETC) activity. Panel **(B)** highlights reduced OXPHOS capacity in left ventricular (LV) myocardial mitochondria, characterized by diminished respiratory complex (I, II, IV, V) activities and coenzyme Q10 depletion in patients with LV systolic dysfunction. Panel **(C)** summarizes alterations in mitochondrial ultrastructure (e.g., impaired respiratory chain organization) and their functional consequences.

As a high-energy-demand organ, the heart relies on diverse metabolic substrates to sustain normal ATP production (15). Accumulating evidence indicates that mitochondrial dysfunction is a critical etiological factor in CVDs. Such dysfunction elevates reactive oxygen species (ROS) levels via oxidative stress, impairing OXPHOS and disrupting energy metabolism, as illustrated in **Figure 2** (16).

The OXPHOS system comprises complexes I–IV and ATP synthase (complex V). Complex I (NADH dehydrogenase) consists of over 40 subunits encoded by both mitochondrial and nuclear genomes. Complex II (succinate dehydrogenase), a nexus between the TCA cycle and the ETC, is entirely nuclear-encoded. Complexes I, III, and IV form supercomplexes to enhance electron transfer efficiency, whereas complex II operates independently. Electrons are transferred via coenzyme Q and cytochrome c to complex IV, establishing a transmembrane proton gradient that drives ATP synthesis by ATP synthase. This process requires coordinated regulation by mitochondrial and nuclear genomes, facilitated by molecular chaperones (e.g., LYRM proteins) and acyl carrier proteins (ACPs), which are essential for subunit assembly and cofactor activation (17).

OXPHOS impairment triggers metabolic reprogramming in upstream pathways to compensate for ATP homeostasis and mitigate ROS production. Dysfunctional metabolic pathways often arise from abnormal accumulation of intermediates, which deplete essential cofactors or activate feedback inhibition mechanisms. Fluctuations in cofactor concentrations and intermediate metabolites further induce post-translational modifications, modulating the activity of metabolic-dependent signaling pathways (e.g., mTOR or AMPK) to drive transcriptional adaptations (18).

In HF patients, mitochondrial ultrastructure and function are frequently altered, including reduced respiratory chain enzyme (complexes I–IV) activity and diminished OXPHOS capacity, though these changes may manifest only in advanced disease stages (17). Stride et al. demonstrated reduced OXPHOS capacity in left ventricular (LV) mitochondria in patients with severe LV systolic dysfunction (LVSD) compared to non-LVSD controls. LVSD myocardium exhibited downregulation of core OXPHOS proteins (e.g., key ETC subunits and ATP synthase components), with significant reductions in complexes I, II, IV, and V (excluding complex III) and a >50% decline in coenzyme Q10 levels (19). Wrogemann and Nylen identified mitochondrial Ca²^+^ overload as a key pathogenic factor in OXPHOS defects, linking impaired electron transport to ROS overproduction. Alterations in any ETC complex can disrupt OXPHOS, exacerbating metabolic dysfunction (20–22).

Given the inhibitory effects of mitochondrial Ca²^+^ overload and ROS on OXPHOS, developing safe and effective metabolic or antioxidant interventions targeting this system remains a critical challenge for improving HF therapy.

### 2.3 Regulation of metabolic pathways

**Figure 3:**
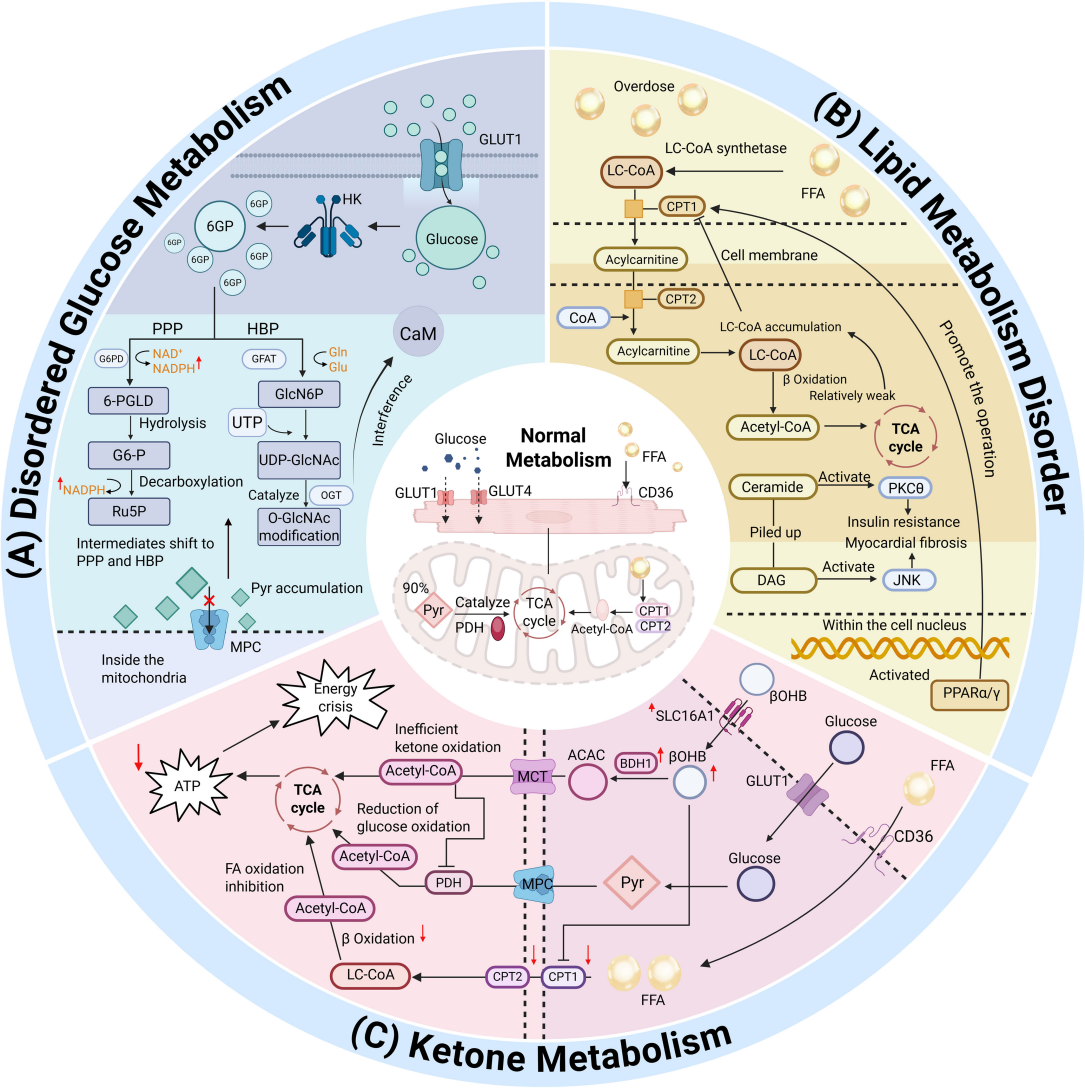
Multimodal regulation of cardiac metabolic flux and enzymatic hierarchy. **(A)** Disordered Glucose Metabolism. **(B)** Lipid Metabolism Disorder. **(C)** Ketone Metabolism. **Figure 3** delineates the multimodal regulation of cardiac metabolic flux and enzymatic hierarchy. Panel **(A)** illustrates abnormal partitioning of glycolytic intermediates under pathological stress, including diversion into the hexosamine biosynthetic pathway (HBP) and pentose phosphate pathway (PPP), driven by impaired mitochondrial pyruvate uptake via MPC1/MPC2 defects. Panel **(B)** depicts lipid overload from PPARα/γ hyperactivation, leading to lipotoxic intermediate accumulation (e.g., ceramides) that activate PKCθ/JNK signaling, insulin resistance, and myocardial fibrosis, alongside endoplasmic reticulum stress (ERS)-mediated regulation of lipid-metabolizing enzymes. Panel **(C)** highlights upregulated ketone utilization in heart failure (e.g., SLC16A1-mediated ketone uptake and BDH1 elevation), despite inefficiencies from low P/O ratios and competition with glucose oxidation.

Cardiac metabolic pathways primarily encompass FA β-oxidation, ketone body metabolism, lactate metabolism, and glycolysis. The flux rates of these pathways are governed by the expression levels of key metabolic proteins (enzymes and transporters) and intricate regulatory mechanisms, as illustrated in **Figure 3**. The transcriptional regulation of enzymes and transporters involved in myocardial energy metabolism remains incompletely understood, reflecting its complexity (2).

#### 2.3.1 Glucose metabolism

Following glucose uptake via GLUT1/GLUT4, abnormal partitioning of glycolytic intermediates significantly influences disease progression. Under physiological conditions, ~90% of pyruvate enters the TCA cycle via pyruvate dehydrogenase (PDH). However, under pathological stress, glycolytic flux increases, diverting intermediates into the hexosamine biosynthetic pathway (HBP) and pentose phosphate pathway (PPP). Fernandez-Caggiano et al. demonstrated that defects in mitochondrial pyruvate carriers 1 and 2 (MPC1/MPC2) suppress mitochondrial pyruvate uptake, driving metabolic reprogramming in the heart and activating PPP to compensate for NADPH deficits via metabolomic profiling (23). While The HBP disrupts calcium regulatory protein function via increased O-GlcNAc glycosylation modifications, the PPP-derived NADPH, despite its antioxidant properties, may paradoxically induce “reductive stress” through excessive activation. This occurs as NADPH overproduction alleviates oxidative damage but concurrently disrupts the NADPH/NADP+ redox equilibrium. Specifically, mitochondrial overproduction of H_2_O_2_ persistently depletes NADPH reserves, triggering compensatory PPP activation to replenish the NADPH pool. However, excessive NADPH accumulation impairs the Keap1-Nrf2 pathway’s adaptive response to oxidative stress and overloads the thioredoxin (Trx) system. This results in hyper-reduction of thiol-dependent proteins, including peroxiredoxins (Prx), thereby compromising their H_2_O_2_-scavenging capacity (24). Such redox dysregulation establishes a vicious cycle that exacerbates mitochondrial oxidative damage while undermining cellular antioxidant defenses.

#### 2.3.2 Lipid metabolism dysregulation

Imbalanced FA β-oxidation and lipotoxicity define lipid metabolic dysregulation. Cardiomyocytes utilize CD36-mediated FA uptake and Carnitine Palmitoyltransferase 1/2(CPT1/2)-regulated mitochondrial transport to convert FAs into acetyl-CoA for the TCA cycle. However, in diabetic or obese states, hyperactivation of PPARα/γ signaling pathways causes lipid overload. When mitochondrial oxidative capacity is overwhelmed, lipotoxic intermediates (e.g., ceramides, diacylglycerols) activate PKCθ and JNK signaling, promoting insulin resistance and myocardial fibrosis. Finck et al. reported that αMHC-PPARα mice fed a long-chain FA diet developed severe lipotoxic cardiomyopathy, which was ameliorated upon switching to a medium-chain triglyceride (MCT)-enriched diet (25). In a subsequent study, systemic CD36 knockout or cardiac-specific LpL deletion in PPARα-transgenic mice reduced lipid uptake and ameliorated cardiomyopathy phenotypes (26). Endoplasmic reticulum stress (ERS) critically modulates this process via IRE1(Inositol-Requiring Enzyme 1)-XBP1(X-box Binding Protein 1) and PERK(Protein kinase R-like Endoplasmic Reticulum Kinase)-eIF2α(Eukaryotic Initiation Factor 2 alpha) pathways, which regulate lipid-metabolizing enzymes while exacerbating myocardial injury through CHOP(CCAAT/Enhancer-Binding Protein Homologous Protein)-mediated apoptosis (27). Recent studies have shown that disorders of mitochondrial lipid metabolism significantly aggravate cardiac systolic dysfunction by promoting acetylation of dynamin−related protein 1 (Drp1). Utilizing a high-fat diet (HFD) murine model and palmitate-treated *in vitro* cardiomyocyte experiments, Hu et al. demonstrated that lipid overload induces decreased intracellular NAD^+^ levels and acetyl-CoA accumulation in cardiomyocytes, thereby triggering acetylation of Drp1 at the lysine 642 (K642) site. This post-translational modification facilitates Drp1 phosphorylation, oligomerization, and mitochondrial translocation, enhances its GTPase activity, and ultimately leads to excessive mitochondrial fission. These pathological alterations result in cardiomyocyte apoptosis, impaired contractile function, and cardiac dysfunction (28).

#### 2.3.3 Ketone body metabolism in heart failure

Emerging studies indicate a complex interplay between ketone metabolism and HF. In HF with reduced ejection fraction (HFrEF), cardiomyocytes upregulate SLC16A1 to enhance ketone uptake, with β-hydroxybutyrate (βOHB) oxidation rates markedly elevated. Aubert et al. revealed through proteomic and metabolomic analyses that HF mice exhibit downregulated mitochondrial FA oxidation proteins but upregulated BDH1, a key ketolytic enzyme, suggesting a metabolic shift favoring ketone utilization over FA oxidation (29). However, the low P/O ratio of ketone oxidation implies inefficiency, and competition with glucose oxidation may exacerbate energy deficits.

## 3 Role of metabolic homeostasis in cardiovascular diseases

The maintenance of energy metabolic homeostasis plays a central role in the pathogenesis and progression of CVDs. Dysregulation of this equilibrium drives cardiac and vascular pathology through mechanisms such as mitochondrial dysfunction, altered substrate preference, and metabolic pathway reprogramming. Whether manifested as the vicious cycle of glucolipid metabolic decompensation and insulin resistance in HF or the glycolysis-driven inflammatory cascade in atherosclerosis, these processes underscore that multidimensional dysregulation of the metabolic regulatory network represents a shared pathological foundation of CVDs.

### 3.1 Heart failure and myocardial infarction

HF and myocardial infarction (MI), as critical conditions in cardiovascular diseases, are both associated with mitochondrial dysfunction and metabolic dysregulation. HF is characterized by impaired left ventricular systolic and diastolic function (10), while MI results from acute myocardial ischemia leading to cardiomyocyte necrosis (30). Both conditions ultimately progress to irreversible cardiac dysfunction through myocardial remodeling (31).

At the level of energy metabolism, HF and MI share common pathological mechanisms, notably reduced mitochondrial oxidative capacity, causing insufficient energy supply. In HF progression, downregulation of myocardial FA oxidation is accompanied by compensatory enhancement of glucose metabolism. Although this adaptation temporarily alleviates energy crisis in the short term, chronic metabolic preference shifts (e.g., suppressed FA oxidation and compensatory upregulation of glucose oxidation) may induce insulin resistance and loss of metabolic flexibility (32). Similar metabolic reprogramming is observed in MI-related ischemia-reperfusion injury (IRI): oxygen deprivation during ischemia forces glycolysis to dominate energy production, while reperfusion reactivates specific metabolic pathways (e.g., succinate accumulation) that exacerbate mitochondrial oxidative stress and ROS bursts, further amplifying myocardial damage (33). Nikolaidis et al. demonstrated that impaired insulin signaling pathways directly correlate with the loss of metabolic flexibility in HF (34), while Dávila-Román et al. confirmed through clinical studies that HF patients exhibit suppressed FA metabolism and enhanced glucose utilization in the myocardium (35). Similarly, Young et al. observed accelerated GLUT-1/4 membrane translocation promoting glycolysis in ischemic regions of MI models, though severe ischemia may worsen injury due to lactate accumulation or excessive ROS production (36).

Intervention strategies targeting metabolic pathways show therapeutic potential for both diseases. Bersin et al. demonstrated that sodium dichloroacetate, a metabolically targeted agent, improved cardiac function in HF patients by optimizing substrate utilization, underscoring the translational potential of metabolic (35). In MI, targeting glycolysis, succinate metabolism, or mitochondrial ROS (e.g., MitoQ-mediated mitochondrial ROS scavenging (37) reduces infarct size and mitigates injury. Notably, metabolic alterations in HF exhibit chronic adaptive features, whereas metabolic dysregulation in MI predominantly manifests as acute decompensation, leading to differences in therapeutic time windows and target selection between the two conditions.

Critical unresolved scientific questions include elucidating the mechanistic role of metabolic reprogramming across disease stages (34), defining the temporal regulatory patterns of key metabolic nodes (e.g., glycolysis, ketone metabolism, succinate signaling) (33, 36), and optimizing clinical translation strategies for metabolic modulators (37, 38). Addressing these challenges will drive the development of novel therapeutic paradigms in “metabolic cardiology,” bridging fundamental discoveries to clinical applications.

### 3.2 Atherosclerosis

Atherosclerosis, a disease characterized by chronic vascular inflammation and lipid deposition, drives the development of cardiovascular, cerebrovascular, and peripheral vascular pathologies through plaque formation. Sarrazy et al. demonstrated in an *Apo E* ^−/−^ murine atherosclerosis model that hematopoietic stem cell-specific *Glut1* deletion significantly suppressed glycolytic activity, identifying Glut1-mediated glycolysis as a pivotal metabolic driver of atherosclerosis (39). Recent studies have revealed a critical association between ferroptosis and the progression of atherosclerosis, with its core mechanisms involving lipid peroxidation and dysregulated glutathione metabolism. Specifically, excess iron in intraplaque macrophages enhances oxidative stress through the Fenton reaction, triggering the release of inflammatory cytokines and thereby accelerating plaque destabilization and thrombosis risk (38). These findings position ferroptosis as a pivotal therapeutic target for modulating plaque stability in atherosclerotic CVD. Inflammation is another hallmark of atherosclerosis, with macrophages serving as primary inflammatory mediators. Glycolytic intermediates in macrophages regulate pro-inflammatory cytokine release, suggesting that targeting glycolysis may modulate atherosclerotic inflammation. Yamashita et al. employed a rabbit atherosclerosis model coupled with metabolomics to reveal that hypoxia amplifies intraplaque macrophage glycolysis via the HIF-1α pathway, exacerbating plaque inflammation and thrombotic risk (40).

The endothelium, a critical regulator of vascular homeostasis, undergoes functional impairment early in atherogenesis. Endothelial cells, which rely predominantly on glycolysis due to their sparse mitochondrial content (41), activate pro-inflammatory pathways and upregulate glycolytic enzymes in atherosclerotic regions. Inhibition of PFKFB3 (6-phosphofructo-2-kinase/fructose-2,6-biphosphatase 3), a key glycolytic enzyme, suppresses inflammation-associated angiogenesis, positioning endothelial metabolism as a therapeutic target. Schoors et al. reported that 3PO-mediated PFKFB3 inhibition reduced endothelial glycolysis and angiogenesis (42). However, current research disproportionately focuses on PFKFB3, with limited exploration of other rate-limiting glycolytic enzymes (e.g., HK, PK). Contradictory findings, such as the lack of plaque progression attenuation upon myeloid-specific PFKFB3 inhibition, underscore the cell type-dependent roles of glycolysis in atherosclerosis.

### 3.3 Pulmonary arterial hypertension

Pulmonary arterial hypertension (PAH), a disease with high morbidity and mortality, is characterized by progressive pulmonary vascular obstruction, ultimately leading to right ventricular failure and death. Growing evidence implicates systemic metabolic dysregulation as both a correlate and potential driver of PAH pathogenesis (43). Pharmacological activation of pyruvate dehydrogenase (PDH) via dichloroacetate (DCA), a pyruvate dehydrogenase kinase (PDK) inhibitor, has been shown to reverse PAH symptoms, underscoring that targeting mitochondrial metabolism directly ameliorates vascular hyperproliferation and apoptosis resistance in PAH (44).

In PAH, inhibition of PDH redirects pyruvate conversion to lactate via lactate dehydrogenase A (LDHA), resulting in reduced ATP production compared to OXPHOS—a phenomenon termed the Warburg effect. This metabolic shift drives pulmonary arterial vascular cells to adopt a cancer-like phenotype characterized by hyperproliferation and apoptosis resistance. Recent work by Sun et al. further elucidates that mitochondrial hyperfission increases mitochondrial reactive ROS(mtROS), particularly H_2_O_2_, through a Drp1-dependent mechanism. These mtROS oxidize the Cys³²^6^ residue of prolyl hydroxylase domain-containing protein 2 (PHD2), inducing its dimerization-mediated inactivation. Consequently, hypoxia-inducible factor-1α (HIF-1α) is stabilized, upregulating glycolysis-promoting enzymes such as LDHA and exacerbating metabolic reprogramming (45). Notably, mitochondrial ROS critically regulate HIF-1α expression. Patten et al. demonstrated that inhibiting mitochondrial complex III markedly reduces HIF-1 accumulation in vascular smooth muscle cells, establishing mtROS as essential mediators of HIF-1 stabilization and transcriptional activation even under normoxic conditions (46). This redox-metabolic crosstalk underscores the central role of mitochondrial dynamics and ROS signaling in sustaining the pathological metabolic phenotype of PAH.

Right ventricular (RV) failure in PAH patients correlates with mitochondrial metabolic abnormalities. During RV hypertrophy, myocardial hypoxia induces metabolic reprogramming characterized by suppressed mitochondrial glucose oxidation with compensatory reliance on inefficient glycolysis, accompanied by impaired FA β-oxidation. This process is regulated through pyruvate dehydrogenase kinase (PDK) activation and transcription factors (e.g., HIF-1α/Myc), ultimately leading to insufficient ATP synthesis, acidosis, and cardiac functional deterioration (47). Emerging metabolomic evidence reveals systemic FA metabolic dysregulation in PAH patients’ blood and RV tissues: elevated plasma carnitine metabolites indicate mitochondrial FA oxidation impairment (48), while SPECT imaging demonstrates significantly reduced RV FA uptake in severe cases (49). These metabolic defects form a vicious cycle with an accumulation of lipotoxic substances like ceramides (C16:0/C24:1), exacerbating cardiac dysfunction (50). Experimental PDK inhibitors (e.g., dichloroacetate) and FA oxidation modulators show potential to restore glucose metabolic balance and reduce lipotoxicity, thereby improving RV performance. These findings not only establish a direct association between metabolic markers and mitochondrial dysfunction, but also provide a mechanistic rationale for therapeutic strategies targeting PDK activity or metabolic equilibrium, demonstrating significant potential for reversing PAH-associated RV failure.

### 3.4 Diabetic cardiomyopathy

Diabetic cardiomyopathy (DCM) denotes structural and functional cardiac abnormalities arising in the context of diabetes mellitus, which may ultimately progress to HF and contribute to patient mortality. Ferroptosis has emerged as a critical mechanism in DCM. Hyperglycemia is hypothesized to induce myocardial lipid peroxidation via mitochondrial iron overload and suppression of glutathione peroxidase 4 (GPX4) activity, leading to impaired myocardial contractile function (51). These findings underscore the interplay between iron-dependent oxidative damage and metabolic dysregulation in driving diabetic cardiac pathology, highlighting ferroptosis as a potential therapeutic target for mitigating diabetes-associated cardiac dysfunction. Although glycemic control and conventional HF therapies provide partial benefits, no disease-modifying treatments specifically targeting DCM currently exist. Diabetes perturbs myocardial substrate utilization, promoting excessive FA oxidation and suppressing glycolysis. ROS-induced mitochondrial uncoupling in diabetic hearts markedly reduces cardiac efficiency, while advanced glycation end-product (AGE) accumulation and dysregulated HBP activity exacerbate glucotoxicity.

Over recent decades, extensive research has elucidated DCM pathophysiology, with mounting evidence implicating impaired mitochondrial energy metabolism as a central pathogenic mechanism (42). For instance, Ni et al. demonstrated in streptozotocin (STZ)-induced type 1 diabetic mice that the mitochondria-targeted antioxidant mito-TEMPO significantly attenuates myocardial oxidative damage and improves diastolic function, indicating mitochondrial ROS as a critical therapeutic target in DCM (52). The imbalance between excessive ROS production and diminished detoxification capacity drives oxidative injury. Impaired insulin signaling itself profoundly impacts mitochondrial morphology and function. Using cardiomyocyte-specific insulin receptor knockout (CIRKO) mice, Boudina et al. revealed that disrupted cardiac insulin signaling induces mitochondrial metabolic dysfunction and heightened oxidative stress (53). Systemic insulin resistance, a hallmark of diabetes, manifests in multiple tissues—including the heart—in type 2 diabetic models. The team led by E. Dale Abel confirmed via perfusion experiments that ob/ob mice exhibit defective insulin-stimulated glucose uptake and oxidation in cardiomyocytes, establishing insulin resistance as a key mechanism underlying impaired cardiac glucose metabolism in diabetes (54).

## 4 Therapeutic interventions targeting cardiac energy metabolism

**Figure 4:**
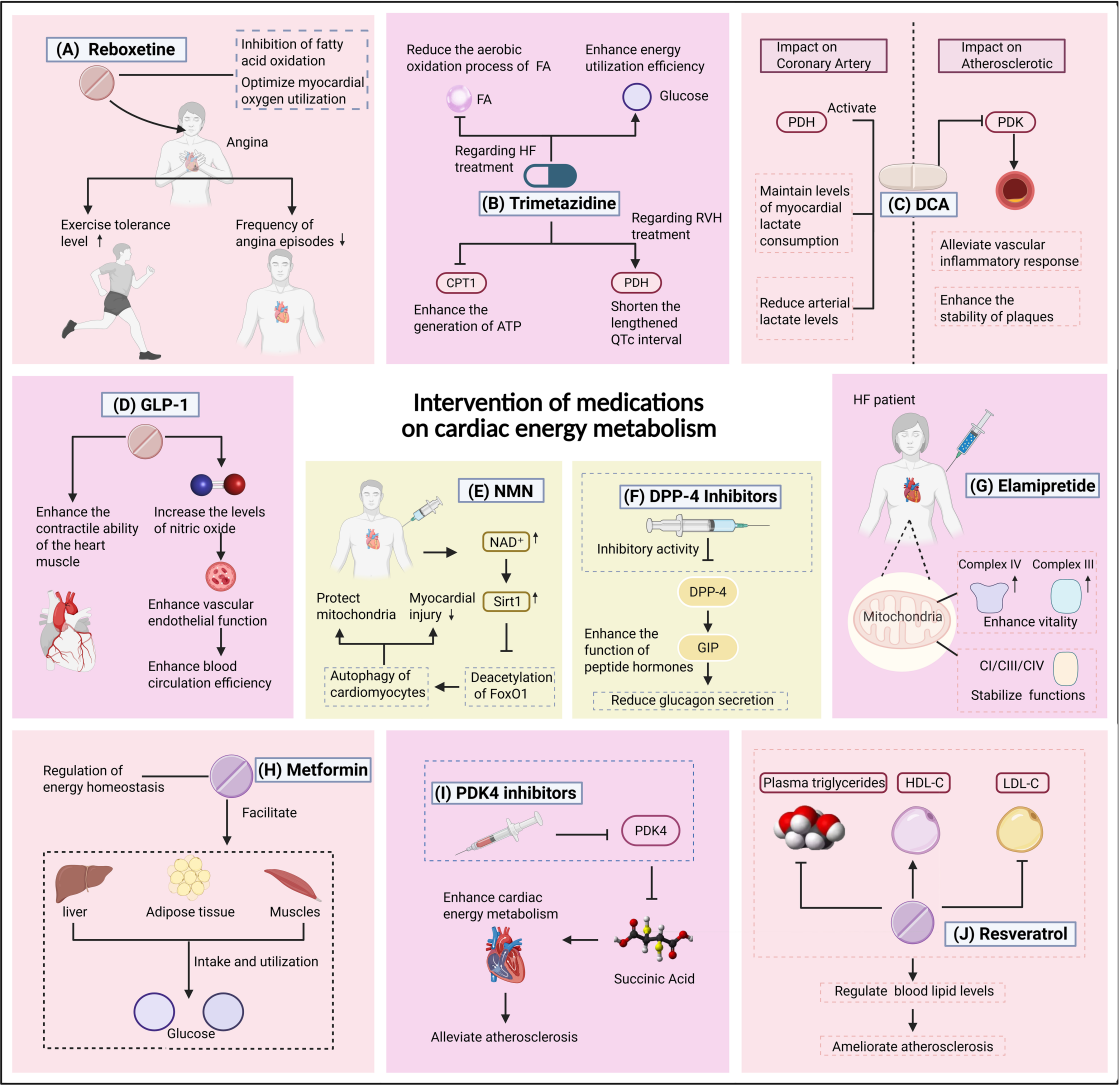
Metabolic-targeted therapeutic interventions for cardiac functional recovery. **(A)** Ranolazine. **(B)** Trimetazidine. **(C)** Dichloroacetate (DCA). **(D)** Glucagon-Like Peptide-1 (GLP-1). **(E)** Nicotinamide Mononucleotide (NMN). **(F)** Dipeptidyl Peptidase-4 (DPP-4) Inhibitors. **(G)** Elamipretide. **(H)** AMPK Activators AMPK Activators. **(I)** Pyruvate Dehydrogenase Kinase 4 (PDK4) Inhibitors. **(J)** Resveratrol (RE). **Figure 4** outlines metabolic-targeted therapeutic interventions for cardiac functional recovery. Panel **(A)** (Ranolazine) highlights its inhibition of fatty acid oxidation (FAO) to optimize myocardial oxygen utilization and alleviate angina symptoms. Panel **(B)** (Trimetazidine) emphasizes suppression of CPT1-mediated FA transport and enhanced pyruvate dehydrogenase (PDH) activity to shift substrate utilization toward glucose. Panel **(C)** (Dichloroacetate/DCA) illustrates PDH activation to promote pyruvate oxidation and improve lactate clearance. Panel **(D)** (GLP-1) focuses on enhancing myocardial contractility and circulation efficiency through metabolic regulation. Panel **(E)** (NMN) depicts NAD^+^ biosynthesis to restore mitochondrial homeostasis and activate Sirt1-dependent cardioprotective pathways. Panel **(F)** (DPP-4 inhibitor) showing enhanced peptide hormones function and increased insulin secretion. Panel **(G)** (Elamipretide) stabilizes mitochondrial supercomplexes (CI/CIII/CIV) to enhance OXPHOS efficiency. Panel **(H)** (Metformin) promotes the uptake and utilization of glucose by the liver, adipose tissue and muscles to regulate energy balance. Panel **(I)** (PDK4 Inhibitors) (PDK4 inhibitor) targets PDK4 to improve energy metabolism to alleviate atherosclerosis. Panel **(J)** (Resveratrol/RE) emphasizes antioxidant and anti-inflammatory effects to regulate blood lipids and inhibit atherosclerosis.

In response to the pathological features of cardiac metabolic dysregulation, multifaceted intervention strategies have been developed in both clinical and research settings. These approaches target critical nodes in energy metabolism—including FAO, glucose utilization, mitochondrial function, and metabolic reprogramming—to restore cardiac function through distinct pharmacological mechanisms. Key therapeutic modalities are summarized in **Figure 4**.

### 4.1 Fatty acid oxidation inhibitors

#### 4.1.1 Ranolazine

Chaitman et al. demonstrated in the Monotherapy Assessment of Ranolazine In Stable Angina (MARISA) trial—a randomized, double-blind, crossover study—that ranolazine monotherapy significantly improved exercise tolerance, delayed ischemia onset, and enhanced long-term survival in patients with chronic severe angina. Emerging evidence suggests that ranolazine may optimize myocardial oxygen metabolic balance by selectively inhibiting the late sodium current, thereby reducing intracellular calcium overload in cardiomyocytes, improving diastolic function, and decreasing arrhythmia susceptibility (55–58). These pleiotropic effects position ranolazine as a promising therapeutic agent for addressing both metabolic and electrophysiological dysregulation in CVDs.

These benefits are mechanistically linked to its inhibition of FA oxidation and optimization of myocardial oxygen utilization (59). While the precise molecular mechanisms of ranolazine remain incompletely elucidated, multiple clinical studies confirm its superiority over placebo in improving exercise capacity and reducing angina frequency. Ranolazine may also be combined with beta-blockers or calcium channel blockers to augment therapeutic efficacy (60). The Ranolazine Open Label Experience (ROLE) study by Koren et al., which followed patients with severe chronic stable angina, further highlighted ranolazine’s favorable long-term tolerability and safety profile (61). However, Salazar et al. cautioned that while ranolazine as adjunctive therapy reduces angina episodes, its clinical application requires careful consideration of non-serious adverse event risks, and the benefits of ranolazine monotherapy remain inadequately defined (62).

#### 4.1.2 Trimetazidine

Trimetazidine exerts cardioprotective effects by mitigating cardiomyocyte apoptosis, fibrosis, autophagy, and inflammatory responses. Numerous clinical studies consistently demonstrate that adjunctive trimetazidine therapy improves symptoms in HF patients when combined with standard treatments (63). This agent reduces aerobic FAO and promotes glucose utilization, thereby enhancing energy efficiency through metabolic substrate switching. Elevated lactate levels, strongly correlated with HF severity and poor prognosis, are significantly higher in HF patients compared to healthy individuals (64–67). Using metabolomic and functional assays, Fang et al. confirmed that trimetazidine effectively reverses metabolic imbalance during right ventricular hypertrophy (RVH) by suppressing CPT1 expression and enhancing PDH activity. This mechanism not only boosts ATP production but also normalizes prolonged QTc intervals, establishing trimetazidine as a viable strategy for managing right ventricular failure (68).

#### 4.1.3 Dichloroacetate

Dichloroacetate (DCA) enhances pyruvate oxidative metabolism by activating PDH, favoring carbohydrate oxidation over FA pathways. It also improves lactate metabolism, facilitating the clearance of lactate accumulated during myocardial ischemia (69). Wargovich et al. demonstrated in coronary artery disease patients that DCA activates PDH, optimizes myocardial metabolic efficiency, reduces arterial lactate levels, and enhances cardiac work efficiency via increased stroke volume and decreased systemic vascular resistance (70). Forteza et al. identified the PDK1/PDH axis as a critical mediator of vascular inflammation and plaque instability in atherosclerosis. DCA counteracts these effects by inhibiting PDK, attenuating inflammation, and stabilizing plaques (71). Thus, DCA-based therapies targeting the PDK/PDH axis hold promise for reshaping the immunometabolic microenvironment and halting atherosclerosis progression.

### 4.2 Glucose metabolism modulators

#### 4.2.1 Glucagon-like peptide-1

Glucagon-like peptide-1 (GLP-1) not only regulates glycemic control but also exerts cardiovascular protective effects through multiple non-glycemic mechanisms. These include anti-atherosclerotic actions (e.g., inhibition of vascular smooth muscle cell proliferation and matrix metalloproteinase-2 [MMP-2] activity), improvement of endothelial function (via nitric oxide [NO]-mediated vasodilation and reduced oxidative stress), and anti-inflammatory effects (72). These pleiotropic properties highlight GLP-1’s potential as a therapeutic agent for addressing both metabolic and vascular pathologies in CVDs. Almutairi et al. demonstrated in animal models that GLP-1 enhances myocardial contractility (73). It also improves endothelial function by elevating nitric oxide levels, promoting vasodilation, and mitigating ischemic injury (74). Clinical evidence highlights GLP-1’s benefits in HF patients, notably associated with improvements in left ventricular ejection fraction (LVEF)—a key indicator of systolic function. For instance, GLP-1 infusion or analogs significantly increase LVEF in HF with reduced ejection fraction (HFrEF), suggesting dual roles in metabolic regulation and contractility enhancement (75, 76). However, the precise pathways underlying GLP-1’s inotropic effects require further elucidation.

#### 4.2.2 Dipeptidyl peptidase-4 inhibitors

Dipeptidyl peptidase-4 (DPP-4) inhibitors (gliptins) augment insulin secretion and suppress glucagon by prolonging the activity of incretins like GLP-1 and GIP (77). Despite their theoretical benefits, clinical outcomes in HF are mixed. While the TECOS trial reported neutral cardiovascular effects for sitagliptin, other agents like saxagliptin are associated with increased HF hospitalization risk (78). These disparities underscore the importance of individualized therapeutic strategies in HF management. The differential effects of saxagliptin and sitagliptin on HF risk likely reflect their distinct pharmacodynamic mechanisms. Studies suggest that saxagliptin may disrupt myocardial energy metabolism by inhibiting sodium-glucose cotransporter (SGLT1/2)-dependent glucose uptake in cardiomyocytes, a phenomenon not observed with sitagliptin. Additionally, saxagliptin-mediated dipeptidyl peptidase-4 (DPP-4) inhibition elevates circulating stromal cell-derived factor-1α (SDF-1α), potentially exacerbating myocardial fibrosis and inflammatory responses (79). Variations in substrate selectivity among DPP-4 inhibitors may further influence cardiovascular outcomes, while patient population heterogeneity—through altered pharmacokinetics or pharmacodynamics—could amplify these disparities. These findings underscore the imperative of developing personalized therapeutic strategies tailored to HF patients.

### 4.3 Mitochondria-targeted agents

#### 4.3.1 Elamipretide

Elamipretide (Bendavia/MTP-131/SS-31), a cell-penetrant aromatic-cationic tetrapeptide, represents a novel class of mitochondria-targeted therapeutics (80, 81). In animal models, elamipretide rapidly accumulates in tissues, elevates energy production, reduces ROS, and protects against IRI. Sabbah et al. demonstrated in advanced HF dogs that chronic subcutaneous elamipretide administration optimizes mitochondrial energy metabolism, with efficacy comparable to that of ACE inhibitors or β-blockers. This positions elamipretide as a complementary strategy to conventional afterload-reducing therapies (73). Chatfield et al. further validated its clinical potential in human HF, showing that elamipretide stabilizes mitochondrial supercomplexes (CI/CIII/CIV), enhances complex I/IV activity, and improves energy metabolism ex vivo, with a favorable safety profile (71).

#### 4.3.2 Nicotinamide mononucleotide

Cao et al. confirmed in a murine sepsis model that nicotinamide mononucleotide (NMN), as a central molecule in energy metabolism, exerts critical organ-protective effects in sepsis by regulating mitochondrial homeostasis and immune cell function. This finding suggests its potential therapeutic value in cardiovascular diseases (CVDs), where NMN may ameliorate energy metabolism disorders and inflammatory injury through analogous mechanisms (82).Nicotinamide mononucleotide (NMN), a biosynthetic precursor of the mitochondrial energy metabolism-essential molecule NAD^+^, demonstrates therapeutic potential in attenuating the progression of CVDs such as HF and atherosclerosis, primarily via its role in NAD^+^ biosynthesis. Multiple clinical studies have investigated NMN’s efficacy and safety, revealing its promising cardiovascular protective effects without significant adverse events (83, 84). For instance, Yamamoto et al. demonstrated that NMN supplementation preserves cardiomyocyte NAD^+^ levels, activates Sirt1-dependent FoxO1 deacetylation, and enhances autophagy, thereby protecting mitochondrial function and mitigating ischemia-reperfusion-induced myocardial injury (85). Similarly, Zhang et al. reported that short-term NMN administration restores cardiac NAD^+^ content, reduces mitochondrial protein hyperacetylation, and enhances FAO capacity, effectively maintaining mitochondrial homeostasis to prevent pressure overload-induced HF (86). These findings highlight NMN’s capacity to modulate mitochondrial bioenergetics and redox balance, identifying NMN as a novel therapeutic candidate for targeting metabolic dysregulation in CVDs.

### 4.4 Metabolic reprogramming agents

#### 4.4.1 AMPK activators

AMPK, a central regulator of energy homeostasis, coordinates metabolic pathways to balance energy supply and demand. Metformin, a well-established AMPK activator, specifically inhibits mitochondrial complex I. This agent enhances glucose uptake and utilization in the liver, muscle, and adipose tissue, serving as a first-line therapy for type 2 diabetes mellitus (T2DM) while conferring cardiovascular benefits independent of its hypoglycemic effects (74, 87). Despite extensive research, the precise molecular mechanisms underlying metformin’s cardio protection remain incompletely elucidated. In DCM—a severe complication characterized by cardiac metabolic dysregulation, exacerbated inflammation, fibrosis, and progressive cardiac dysfunction—activation of the NLRP3 inflammasome drives pyroptosis and inflammatory cascades. Using streptozotocin-induced diabetic mouse models and high glucose-treated cardiomyocytes, Yang et al. demonstrated that metformin activates AMPK via complex I inhibition, subsequently suppressing mTOR signaling, enhancing autophagy, and attenuating inflammasome-mediated pyroptosis and inflammation. These findings provide direct evidence for metformin’s cardioprotective pleiotropic mechanisms in diabetic cardiovascular complications (88).

#### 4.4.2 Pyruvate dehydrogenase kinase 4 inhibitors

Pyruvate dehydrogenase kinase 4 (PDK4) critically regulates glucose and FA metabolism by inhibiting PDH activity. Accumulating evidence links PDK4 overexpression or hyperactivation to myocardial injury and cardiac dysfunction, while PDK4 inhibition optimizes cardiomyocyte energy metabolism and protects against cardiac damage (89). Forteza et al. integrated clinical data with metabolomics to reveal that elevated PDK4 expression correlates with plaque metabolic dysregulation in symptomatic carotid artery disease, characterized by succinate accumulation. This positions PDK4 as a promising therapeutic target to mitigate atherosclerosis progression (71). In a transverse aortic constriction (TAC)-induced HF mouse model, Aizawa et al. evaluated a novel PDK4 inhibitor, demonstrating its efficacy in improving HFrEF-associated dysfunction through metabolic modulation. These findings highlight PDK4 inhibition as an emerging and innovative strategy for CVD management (89).

### 4.5 Emerging metabolic modulators

#### 4.5.1 Resveratrol

Resveratrol (RE), a natural stilbenoid polyphenol, improves endothelial function, scavenges ROS, attenuates inflammation, inhibits platelet aggregation, ameliorates lipid profiles, and counteracts other pro-atherogenic factors (90–92). Given the pivotal role of low-density lipoprotein (LDL) in atherogenesis, RE’s lipid-modulating effects hold therapeutic significance. Yeşim et al. reported that RE reduces plasma triglycerides and LDL while elevating high-density lipoprotein (HDL) levels (93). Additionally, RE upregulates LDL receptor expression in hepatocytes, further lowering circulating LDL (94). Its antioxidant properties not only reduce LDL oxidation but also activate endogenous antioxidant systems, exerting anti-inflammatory effects. RE’s inhibition of smooth muscle cell migration further contributes to its anti-atherogenic potential (59, 94, 95). Collectively, these properties underscore RE’s potential as a multi-targeted therapeutic agent against the multifactorial pathology of atherosclerosis.

## 5 Discussion

Energy metabolism, as a core process of cellular life activities, involves intricate molecular mechanisms and regulatory networks. It has long been widely accepted in the scientific community that its primary functions revolve around ATP generation and nutrient catabolism. However, with advancing research, energy metabolism is now recognized not merely as an energy supply system but as a critical regulator of cellular signaling, epigenetic modulation, and cell cycle control. These groundbreaking insights have reshaped traditional perspectives on energy metabolism and opened new avenues for diagnosis and treatment of metabolic disorders. (i) Energy metabolism dysregulation plays a crucial role in cardiomyocyte adaptation and repair processes. Taking myocardial remodeling as an example, while traditional perspectives emphasize its association with hemodynamic overload or neurohormonal activation, recent studies reveal that mitochondrial dysfunction exhibits dual pathological mechanisms in CVDs. On one hand, abnormalities in mitochondrial respiratory chain function, diminished ATP synthesis capacity, and elevated oxidative stress collectively form the core pathological basis of cardiomyocyte energy metabolism disruption. Concurrently, defective mitophagy mediated by the PINK1/Parkin pathway exacerbates ,OXPHOS dysfunction. On the other hand, mitochondrial dynamic imbalance—particularly excessive fission-induced mitochondrial fragmentation—interferes with autophagic clearance of damaged mitochondria via mechanisms such as Drp1-Ser616 phosphorylation, identified as a key driver of apoptosis in ischemic myocardial injury. Research demonstrates that the PINK1/Parkin pathway maintains mitochondrial metabolic homeostasis by ubiquitinating damaged mitochondria and recruiting autophagy receptors (e.g., OPTN, NDP52), underscoring its central regulatory role (16, 96, 97). Targeting mitochondrial energy metabolism pathways and restoring mitochondrial dynamic equilibrium may therefore represent novel therapeutic strategies for CVD intervention.(ii) Metabolic intermediates are not merely energy carriers but also act as critical signaling molecules modulating pathophysiological processes. For instance, succinate rapidly accumulates in cardiac tissues during normothermic ischemia, triggering ROS bursts via mitochondrial reverse electron transport, directly exacerbating tissue damage (98, 99). This signaling cascade can be precisely modulated through targeted interventions: strategies such as hypothermic preservation or inhibition of succinate dehydrogenase (e.g., using dimethyl malonate) effectively block succinate accumulation and oxidation, reducing ROS levels and improving transplanted heart function (100). These findings highlight the signaling properties of metabolic intermediates as innovative targets for developing precision metabolic therapies, bridging metabolic flux with redox and inflammatory signaling networks in cardiovascular pathologies. (iii) The systemic interplay between metabolic reprogramming and CVD progression is increasingly recognized. Pathological shifts in cardiomyocyte substrate preference—such as the transition from FA oxidation to glycolysis-dominant energy production—not only exacerbate myocardial energy crises but also directly drive ventricular remodeling by altering cellular phenotypic plasticity (101–103). Recent evidence further reveals that immune cell metabolic reprogramming (e.g., glycolysis-dependent pro-inflammatory polarization of macrophages) modulates inflammatory microenvironments, critically influencing atherosclerotic plaque stability. Such cross-tissue and cross-cellular metabolic network interactions provide novel insights into the multi-organ pathological cascades of CVDs (104, 105).

Despite significant progress in elucidating the role of energy metabolism in cellular function and disease pathogenesis, current research faces several limitations: (i) The interplay between distinct metabolic pathways and their disease-specific regulatory mechanisms remains incompletely defined. While glycolysis, OXPHOS, and FA metabolism have been extensively studied, their synergistic or antagonistic roles in specific pathological contexts require deeper exploration. For example, although metabolic modulators like GLP-1 improve cardiac hemodynamics, their direct metabolic mechanisms remain elusive. Others, such as etomoxir, face clinical translation barriers due to off-target effects like skeletal muscle lipotoxicity (106). (ii) Existing studies predominantly focus on the energy-supplying role of metabolic pathways, overlooking the regulatory impacts of metabolites as second messengers or epigenetic modifiers. TCA cycle intermediates such as succinate and α-ketoglutarate have been implicated in inflammation and fibrosis, yet their precise regulatory networks in cardiomyocyte apoptosis or pathological hypertrophy remain unclear (107). (iii)While emerging studies have established that TCA cycle intermediates (e.g., succinate, α-ketoglutarate) regulate inflammatory responses and fibrotic progression through modulation of HIF-1α stability and histone demethylase activity (108), their roles as oxidative stress signaling hubs in cardiac diseases remain incompletely elucidated. Notably, α-ketoglutarate (α-KG), serves as an essential cofactor for DNA/histone demethylases, suppresses cardiac fibroblast activation. However, systematic investigations into its spatiotemporally specific metabolic regulatory networks governing cardiomyocyte apoptosis resistance and their crosstalk with the AMPK/mTOR signaling axis are still lacking (109). This knowledge gap hinders the development of α-KG-based therapies targeting metabolic-epigenetic interplay in cardiac remodeling and stress adaptation.

Future research should prioritize unraveling the complex relationship between energy metabolism and CVDs: (i) Investigate the detailed mechanisms by which metabolic pathways (e.g., glycolysis, OXPHOS, FA metabolism) regulate cardiomyocyte survival and death, with emphasis on their crosstalk with oxidative stress, mitochondrial function, and cellular signaling. (ii) Integrate metabolomics, epigenomics, and single-cell multi-omics technologies to systematically map the dynamic flux of metabolic intermediates and their non-canonical roles in pathological processes such as myocardial remodeling and apoptosis resistance. (iii) Future research should focus on clarifying the dynamic accumulation patterns of succinate and α-ketoglutarate (α-KG) within the myocardial microenvironment, as well as their non-canonical signaling mechanisms, to advance the development of cardioprotective strategies targeting metabolite-mediated signal transduction. This includes delineating how spatial-temporal fluctuations in these TCA cycle intermediates modulate redox signaling, epigenetic reprogramming, and intercellular crosstalk under pathological stress, thereby bridging metabolic dynamics with adaptive or maladaptive cardiac remodeling processes.

## 6 Conclusion

Energy metabolism dysregulation represents a central pathological mechanism underlying diverse CVDs, characterized by mitochondrial dysfunction and imbalances in glycolysis and FA metabolism. These aberrations not only compromise myocardial energy supply but also exacerbate cardiac functional deterioration through oxidative stress, inflammatory cascades, and other pathological pathways. Previous studies have predominantly focused on isolated metabolic pathways or disease-specific models, lacking a holistic understanding of energy metabolism dysregulation in CVDs. Critical knowledge gaps persist regarding the dynamic evolution of metabolic reprogramming across disease stages and its cross-organ interaction mechanisms. Furthermore, current therapeutic strategies face limitations in clinical translation due to insufficient target specificity and off-target effects, reflecting the unresolved multidimensional complexity of metabolic regulatory networks. This review systematically synthesizes key regulatory nodes in cardiac energy metabolism and their pathological remodeling mechanisms in conditions such as HF, atherosclerosis, and pulmonary arterial hypertension, elucidating the synergistic pathogenic network between metabolite-mediated signaling and mitochondrial dysfunction. Given the limitations of existing interventions, we propose a novel paradigm for precision modulation of metabolic homeostasis. Future research should integrate multi-omics technologies with clinical insights to decode the crosstalk between metabolic pathways and their dynamic regulatory networks, ultimately advancing the development of precision metabolic therapeutics to overcome current treatment barriers and improve patient outcomes.
